# High-Prevalence Vitamin D Deficiency among Korean Emergency Department Homeless, with a Comparison to a Healthy Korean Population

**DOI:** 10.3390/nu11040763

**Published:** 2019-04-01

**Authors:** Hui Jai Lee, Jonghwan Shin, Kyoung Min You

**Affiliations:** 1 Department of Emergency Medicine, Seoul Metropolitan Government Seoul National University Boramae Medical Center, 20, Boramae-ro 5-gil, Dongjak-gu, Seoul 07061, Korea; emdrlee@snu.ac.kr (H.J.L.); ykminnim@gmail.com (K.M.Y.); 2 Department of Emergency Medicine, Seoul National University College of Medicine, 103 Daehak-ro, Jongno-gu, Seoul 03080, Korea

**Keywords:** vitamin deficiency, homeless, hemoglobin, anemia, rhabdomyolysis

## Abstract

Although nutritional problems are a major concern for the homeless, their vitamin D status has not yet been widely evaluated. This study was a retrospective chart review conducted at a single academic, urban public hospital’s emergency department (ED). Patients whose serum 25-hydroxyvitamin D [25(OH)D] levels had been checked in the ED from July 2014 to June 2015 were reviewed and enrolled. For a healthy settled civilian control, 2011 and 2012 data from the Fifth Korean National Health and Nutrition Examination Survey (KNHANES) were used. A total of 179 patients were enrolled. Vitamin D deficiency was observed in 133 patients (73.7%). The vitamin D deficiency group showed a lower hemoglobin level than that of non-vitamin D deficiency group (*p* = 0.02). Winter visits were more common among the deficiency group (*p* = 0.048). Rhabdomyolysis was observed only in the deficiency homeless group (*p* = 0.03). When using age and sex as covariates of propensity score matching 25(OH)D levels were lower in the homeless than in the healthy control (15.7 ± 7.4 ng/mL vs. 18.2 ± 5.5 ng/mL, *p* < 0.001). Moreover, when the controls were limited to residents of the same city, the serum 25(OH)D level also was lower in the homeless than in the control (15.7 ± 7.4 ng/mL vs. 17.1 ± 5.4 ng/mL, *p* = 0.03). In summary, vitamin D deficiency was common and more frequent among homeless patients.

## 1. Introduction

Nutrition deficiencies are among the major problems of the homeless, and adequate nutritional support is essential to their care [1,2,3]. However, macronutrients usually are the major nutritional concerns, with essential micronutrients often being overlooked [4]. Whereas various micronutrients have been evaluated in a few studies, there is still only limited information on micronutrient status among the homeless [3].

Vitamin D is an important micronutrient of bone metabolism. Moreover, recent studies have shown that vitamin D plays important roles in the modulation of immune responses. Vitamin D deficiency is common worldwide, and is associated with fracture, myopathy, cardiovascular disease, diabetes, psychiatric diseases, infections, and critical illness [5,6,7,8,9,10].

Many vitamin-D-deficiency-related clinical conditions such as alcoholism, musculoskeletal injuries and chronic illness also are known to be common among the homeless. Despite the fact that vitamin D deficiency is a significant health problem, the vitamin D status of the homeless has not been evaluated [3].

The purpose of the present study was to assess vitamin D deficiency’s frequency among the homeless and to determine its related clinical conditions.

## 2. Materials and Methods

### 2.1. Study Design

This study was a retrospective chart review conducted at a single academic, urban public hospital with 58,000 annual emergency department (ED) visits. The hospital is located in Seoul (latitude: 37.6° N), Korea. For the purpose of the study, electronic medical-record data covering the period from July 2014 to June 2015 were reviewed. The collected data included demographics, information on underlying diseases, results of laboratory tests, diagnoses and dispositions. The Institutional Review Board of the study hospital approved the study and waived informed consent (IRB No. 20151222/16-2015-174/011).

### 2.2. Study Subjects

Patients who had been treated in the homeless observation area and whose blood 25-hydroxyvitamin D (25(OH)D) results were available were enrolled. In the study hospital, all homeless patients other than those with Emergency Severity Index (ESI) scores of 1 or 2 were treated in the ED’s specialized homeless unit. All ESI 1 and 2 homeless patients were treated in the resuscitation room or critical care unit of our ED in the same way as other ESI 1 and 2 patients without discrimination [11,12]. Emergency medicine faculty and residents evaluated the patients and determined whether laboratory tests and intravenous fluids were needed. In cases where blood samples had been taken, several vitamin levels, including that of serum 25(OH)D, also were checked (Figure S1).

### 2.3. Measurement

All laboratory tests were conducted in the Central Clinical Laboratory of the study hospital. Serum 25(OH)D (ng/mL) levels were measured by radioimmunoassay in the central laboratory of the study hospital. The DIAsource 25OH Vitamin D total-RIA-CT Kit (DIAsource ImmunoAssays S.A., Nivelles, Belgium) was used. We defined vitamin D deficiency as serum 25(OH)D level of less than 20 ng/mL, as based on previous studies [5,13]. Vitamin B1, B6 and C levels were assessed by subjecting blood samples to high-performance liquid chromatography (HPLC) (Perkin-Elmer Series 200). The vitamin B12 levels were determined by electrochemiluminescence immunoassay (Roche E170). Complete blood counts were measured using an automated hematology analyzer (Siemens ADVIA 2120i). Additionally, chemistry tests were performed using an automated chemical analyzer (UniCel DxC 800).

### 2.4. Healthy Control

The Korean National Health and Nutrition Examination Survey (KNHANES) is a nation-wide cross-sectional health survey dataset for assessment of the health-related behavior, health conditions, and nutritional state of the settled civilian non-institutional population by the Korean Center for Disease Control and Prevention (KCDC). Routine 25(OH)D-level measurements of participants were conducted during KNHANES IV (2007–2009) and V (2010–2012). In the present study, we used second- and third-year (2011, 2012) KNHANES V data as our healthy control [13,14]. The serum 25(OH)D concentrations of this cohort were measured by radioimmunoassay using the 25-Hydroxyvitamin D 125I RIA Kit (DiaSorin Inc., Stillwater, MN, USA) [13].

### 2.5. Statistical Analysis

The Shapiro–Wilk test was utilized to evaluate the normality of the continuous variables. The continuous variables were expressed as the mean ± standard deviation (SD) or median (interquartile range (IQR)). The Student’s T test or Mann–Whitney U test was used as appropriate. Categorical variables were presented as a frequency along with the corresponding percentage and were compared using the chi-square test or Fisher’s exact test as appropriate. Log-binomial regression analysis was conducted for calculation of relative risk (RR) for vitamin D deficiency. Subsequently, variables of which the p value was less than 0.1 in the univariate analysis were included in the log-binomial regression model. All of the analyses were performed with SPSS 22 (IBM, Armonk, New York, NY, USA) and R version 3.4.1 (R Foundation for Statistical Computing, Vienna, Austria). A *p* value less than 0.05 was considered to indicate statistical significance.

Age and sex were adjusted for comparison of the serum 25(OH)D levels between the homeless patients and the healthy control (KNHANES data) by 1:2 propensity score matching using the nearest-neighbor matching method. This study was conducted in Seoul, the largest city of Korea; all of the enrolled homeless patients were regarded as Seoul residents. For an additional analysis, therefore, we selected only Seoul residents in the KNHANES data and compared their serum levels to those of the homeless patients after 1:2 propensity-score matching by age and sex.

The seasons considered were spring (March to May), summer (June to August), fall (September to November), and winter (December to February) [13].

## 3. Results

### 3.1. Characteristics of the Homeless 

During the study period, 406 patients were treated in the homeless observation area. There were 138 revisit cases, which were excluded from further analysis. Laboratory tests were not done for 89 patients (Table S1). Levels of 25(OH)D were examined for a total of 179 patients during the study period. Most patients were classified as alcohol-intoxication on initial presentation (154, 86%) (Table 1). Altered mental status was the most common chief complaint (Table 2). A total of 42 of those patients were admitted, 38 to the general ward and 4 to the intensive-care unit.

### 3.2. Level of 25 (OH)D

The median 25(OH)D levels l of the enrolled homeless population was 14.2 (10.7–19.6) ng/mL. Vitamin D deficiency (serum 25(OH)D < 20 ng/mL) was observed in 74% (133) and severe deficiency (25(OH) D < 10 ng/mL) in 18% of cases (33).

### 3.3. Factors Associated with Vitamin D Deficiency

**Figure 1:**
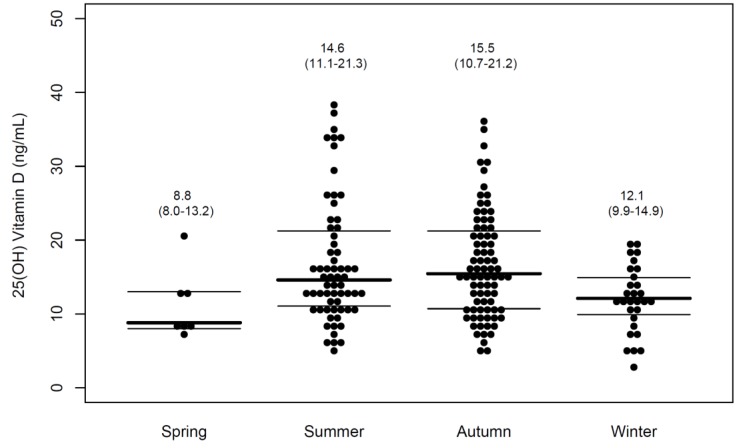
Serum 25(OH)D levels of homeless population according to seasons. The thick lines represent the medians and the thin lines represent the interquartile ranges.

The homeless with vitamin D deficiency had a lower hemoglobin level than that of the non-vitamin-D-deficient homeless (13.4 (12.2–14.8) g/dL vs 14.3 (12.9–15.7) g/dL, *p* = 0.02). Levels were lower in winter and spring than in summer and autumn (*p* < 0.001). In fact, vitamin D deficiencies were common in winter and spring (Figure 1).

The initial creatine kinase (CK) levels did not differ between the groups. However, all of the patients with rhabdomyolysis had decreased 25(OH)D levels. The C-reactive protein levels were higher in the vitamin-D-deficient patient (Table 1). By log-binomial regression analysis, the winter/spring season (RR 5.1, 95% confidence interval (CI) 1.4–17.9) was independently correlated with vitamin D deficiency. Other covariates became non-significant after adjustment (Table 3).

### 3.4. Comparison of Homeless Population with Healthy Controls

For adult participants from the 2011 and 2012 KNHANES V, the mean 25(OH)D level was 17.2 ± 5.7 ng/mL;72.7% had vitamin D deficiencies and 7.1% severe deficiencies.

The homeless patients had a lower 25(OH)D level than that of the healthy national control after propensity matching (15.7 ± 7.4 ng/mL vs. 18.2 ± 5.5 ng/mL, *p* < 0.001).

**Figure 2:**
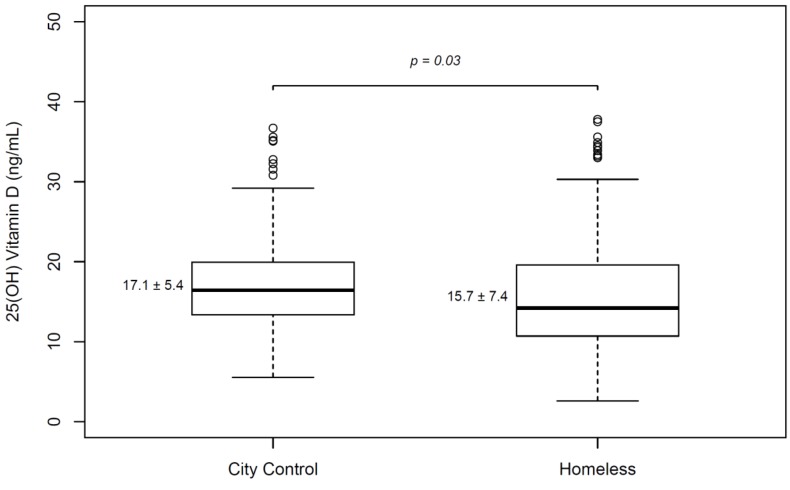
Serum 25(OH)D levels between homeless patients and healthy controls. Homeless patients had lower 25(OH)D levels than same-city controls after 1:2 propensity matching by age and sex. Student’s T test was used.

When the controls were limited to residents of the same city where this study was conducted (Seoul), the serum 25(OH)D level of the homeless also was lower than that of the healthy residents after propensity matching (15.7 ± 7.4 ng/mL vs. 17.1 ± 5.4 ng/mL, *p* = 0.03) (Figure 2) (Tables S2–S5).

## 4. Discussion

Homeless patients have various nutritional problems. They suffer chronic wasting malnutrition due to insufficient food intake, a high proportion of ethanol in their energy intake, as well as chronic illnesses such as diabetes, chronic liver disease, and tuberculosis. Particularly, insufficient intakes of essential micronutrients and combined deficiencies are common. Various kinds of vitamin deficiencies among the homeless have been reported [1,2,3,12].

Many studies have shown high prevalences of vitamin D deficiency worldwide. Inadequate vitamin D intake among the homeless also has been reported [15,16]. Chronic alcohol consumption, which is common among the homeless, increases the risk of vitamin D deficiency. Moreover, vitamin D deficiency also is widespread in chronic pancreatitis, which is a common complication of chronic alcohol consumption [17]. However, few previous studies have evaluated vitamin D levels specifically among homeless people [3].

The results of the present study showed a high prevalence of vitamin D deficiency among the homeless patients. Seasonal variation in blood 25(OH)D levels is well known. As well as the length of sunlight exposure, other factors such as latitude, climate, and culture contribute to the seasonal variation of 25(OH)D levels. Our results indicated that the 25(OH)D levels of winter and spring were lower than those of summer and autumn. Another Korean study of healthy populations also noted lower 25(OH)D levels in winter and spring. However homeless populations were not included in that study [13].

All rhabdomyolysis patients have vitamin D deficiency and are in an acute alcohol-intoxicated state (Table 1). One of the major roles of vitamin D is skeletal action. Vitamin D modulates bone metabolism and also affects the skeletal muscles. The skeletal muscles have a vitamin D receptor, vitamin D being essential for maximal muscle function [5]. Decreased 25(OH)D level is related to impaired muscle function, and supplements have been shown to improve muscle function, especially in the elderly [18]. Moreover, some authors have pointed out an association between vitamin D deficiency and alcohol-induced myopathy [19,20]. The results of our study likewise, showed a possible association between rhabdomyolysis and vitamin D deficiency. However, adjusted analysis failed to show any direct association between vitamin D deficiency and blood CK level or rhabdomyolysis. Further study focusing on rhabdomyolysis and 25(OH)D level among the homeless is required in order to establish the role of vitamin D in alcohol-induced myopathy.

Additionally, we found that the hemoglobin level was lower in the vitamin-D-deficient homeless patients. The results of our multivariate analysis showed that hemoglobin level tended to be positively correlated with vitamin D level, though the association was not statistically significant. Previous studies have demonstrated an anemia-protective function of vitamin D. Immune-modulatory functions such as hepcidin suppression and anti-inflammatory effects have been suggested as being involved in the anemia-protective mechanism of vitamin D [21]. The homeless are exposed to many proinflammatory stresses such as heavy alcohol consumption, smoking, chronic illness, many kinds of antioxidant deficiencies, and decreased immune function [3,12,22]. It is possible that these conditions attenuate the immune modulatory function of vitamin D.

Vitamin D deficiency was more common, and blood levels were lower, in the homeless patients than in the healthy control after adjusting for age and sex. Moreover, when only residents of Seoul (where the study was performed) were selected as the healthy control, the serum 25(OH)D level of the homeless also was lower. Asian studies have shown that urban residents have lower 25(OH)D levels than rural residents [13,23]. In fact, urban residence itself might be a major determinant of vitamin D status among the homeless. However, American studies have shown better vitamin D status for urban residents than for rural residents [24,25]. Other behavior and cultural factors, not evaluated in this study, could also affect vitamin D status.

The homeless have higher morbidity and mortality than the healthy population. They also suffer diabetes, dementia, psychological disorders such as depression, skin diseases and anemia at higher rates. All of these conditions are related and can be worsen by vitamin D deficiency. Moreover, chronic alcohol consumption, other combined chronic illnesses, and many kinds of nutritional deficiencies that are common among the homeless can worsen vitamin-D deficiency-related damage. Vitamin D status should be evaluated for the homeless, and vitamin D supplementation should be considered for vitamin D-deficient homeless people.

Vitamin D deficiencies are common among the healthy Korean population and are increasing. Even in the summer and autumn seasons, mean 25(OH)D levels are just above 20 ng/mL, and most people are at levels classifiable as vitamin D insufficiency [13,26]. The reason is not clear. Ethnicity, genetic properties, and air pollution are possible explanations. Also, vitamin D-fortified food is not popular in Asian countries, and supplementation of micronutrients is often overlooked [27,28,29]. No vitamin D supplementation program or medication was applied for the homeless patients in our study. We think that vitamin D supplementation should be considered for the homeless population of Korea.

There are some limitations to this study. First, because the study was retrospective, nutrient intake patterns, daily activities and sunlight exposure times could not be obtained. Also, information on many clinical factors affecting vitamin D status, such as body mass index (BMI), smoking history, sun exposure time and underlying comorbidity, was not evaluated. Almost all of the patients considered had been in an acutely intoxicated state when admitted to the hospital, and so a detailed medical history taking was problematic, to say the least. Second, severely ill patients (ESI levels 1 and 2) were not included. Moreover, all of the enrolled homeless who had visited the ED had characteristics and vitamin D status that might be different from the healthy homeless. Third, the assay kits used for 25(OH)D measurement between the homeless and the healthy control were different and can contribute to differences in blood 25(OH)D levels. Forth, seasonal data on the control group was not obtained, though 25(OH)D level shows strong seasonal variation. The KCDC, due to privacy concerns, had refused to provide seasonal data from the KNHANES. Fifth, geographic, cultural and behavioral factors affect vitamin D status, and as such, the vitamin D statuses of homeless people in different countries can differ.

All of these limitations notwithstanding, this study is the first to measure the 25(OH)Dlevels of the homeless. To fully account for the limitations above-noted, further multicenter prospective data collection not only from EDs but also homeless care facilities and shelters will be needed.

## 5. Conclusions

Vitamin D deficiency was common among the homeless in our ED. After adjusting for age and sex, the 25(OH)D levels of the homeless were lower than those of a healthy settled civilian control. Vitamin D supplementation should be considered for the treatment of homeless patients. Also, a public health program for the supplementation of vitamin D for homeless populations should be considered.

## Figures and Tables

**Table 1:** Basal characteristics of the homeless.

		Deficient	Not Deficient	*p*-Value *	Total
		(25(OH)D < 20 ng/mL)	(25(OH)D ≥ 20 ng/mL)		
		*n* = 132	*n* = 47		*n* = 179
Age	median (IQR)	53 (46–59)	52 (46–57)		52 (47–58)
Male	*n* (%)	124 (94%)	46 (98%)	0.45	170 (95%)
Alcohol-intoxicated state	*n* (%)	112 (85%)	41 (87%)	0.69	153 (86%)
Past Medical History					
Alcohol dependency	*n* (%)	121 (92%)	44 (94%)	0.67	165 (92%)
Diabetes	*n* (%)	19 (14%)	5 (11%)	0.62	24 (13%)
Hypertension	*n* (%)	14 (11%)	7 (15%)	0.43	21 (12%)
Liver cirrhosis	*n* (%)	12 (9%)	4 (9%)	1.00	16 (9%)
Malignancy	*n* (%)	1 (1%)	1 (2%)	0.43	2 (1%)
Medical conditions					
Active tuberculosis	*n* (%)	4 (3%)	0 (0%) ^†^	0.57	4 (2%)
Infection	*n* (%)	28 (21%)	8 (17%)	0.54	36 (20%)
Trauma	*n* (%)	35 (27%)	12 (26%)	0.90	47 (26%)
Fracture	*n* (%)	7 (5%)	4 (9%) ^†^	0.43	11 (6%)
Rhabdomyolysis	*n* (%)	13 (10%)	0 (0%) ^†^	0.03	13 (7%)
Laboratory test					
WBC (×10^3^/uL)	median (IQR)	7.2 (5.3–9.8)	6.6 (5.6–9.0)	0.55	7.1 (5.4–9.7)
Hb (g/dL)	median (IQR)	13.4 (12.2–14.8)	14.3 (12.9–15.7)	0.02	13.8 (12.5–15.1)
Plt (x10^3^/uL)	median (IQR)	231 (167–278)	219 (137–289)	0.53	229 (155–279)
Sodium (mmol/L)	median (IQR)	139.6 (135.8–142.3)	140.2 (136.6–142.2)	0.49	139.9 (136.3–142.2)
Potassium (mmol/L)	median (IQR)	3.7 (3.4–4.0)	3.7 (3.4–4.0)	0.96	3.7 (3.4–4.0)
Chloride (mmol/L)	median (IQR)	102.4 (96.1–105.5)	102.4 (98.6–105.2)	0.58	102.4 (96.9–105.4)
Calcium (mg/dL)	median (IQR)	8.6 (8.4–9.1)	8.8 (8.4–9.1)	0.44	8.7 (8.4–9.1)
Phosphorus (mg/dL)	median (IQR)	3.5 (2.9–4.3)	3.7 (3.2–4.2)	0.27	3.6 (3.0–4.2)
BUN (mg/dL)	median (IQR)	11 (8–16)	12 (9–16)	0.62	12 (9–16)
Creatinine (mg/dL)	median (IQR)	0.7 (0.6–0.9)	0.8 (0.7–0.9)	0.38	0.7 (0.6–0.9)
AST (IU/L)	median (IQR)	52 (28–104)	44 (27–82)	0.27	50 (27–95)
ALT (IU/L)	median (IQR)	23 (14–50)	24 (16–47)	0.76	23 (15–48)
T.bil (mg/dL)	median (IQR)	1.0 (0.7–1.6)	0.9 (0.7–1.3)	0.51	1.0 (0.7–1.5)
CK (U/L)	median (IQR)	209 (109–453)	203 (122–329)	0.79	208 (114–398)
CK-MB (U/L)	median (IQR)	1.5 (0.8–4.7)	2.5 (1.3–3.65)	0.25	1.9 (0.9–4.3)
Ehtanol (mg/dL)	median (IQR)	220 (2–315)	212 (1–311)	0.64	213 (2–311)
Total protein (g/dL)	median (IQR)	6.7 (6.1–7.1)	6.6 (6.2–7.1)	0.27	6.7 (6.1–7.1)
Albumin (g/dL)	median (IQR)	3.9 (3.6–4.2)	4.1 (3.7–4.3)	0.09	4.0 (3.7–4.2)
Cholesterol (mg/dL)	median (IQR)	164 (134–203)	172 (147–212)	0.47	165 (136–207)
CRP (mg/L)	median (IQR)	0.29 (0.09–0.91)	0.08 (0.03–0.04)	0.005	0.25 (0.05–0.88)
Osmolality (msom/kg)	median (IQR)	343 (297–370)	341 (295–368)	0.75	342.5 (297–370)
Vitamin B1 (mmol/L)	median (IQR)	151 (114–200)	154 (121–226)	0.39	151 (116–205)
Vitamin B12 (pg/mL)	median (IQR)	539 (389–766)	627 (429–936)	0.32	572 (392–804)
Vitamin B6 (mmol/L)	median (IQR)	32.3 (22.1–51.7)	39.2 (22.5–63.0)	0.27	34.1 (22.1–53.3)
Vitamin C (umol/L)	median (IQR)	3.9 (1.3–12.1)	5.4 (1.6–14.7)	0.23	4.1 (1.5–12.7)

* Chi-square test or Mann–Whitney U test were used ^†^ Fisher’s exact test was used. Vit: vitamin, WBC: white blood cell. Hb: hemoglobin, BUN: blood urea nitrogen, AST: aspartate aminotransferase, ALT: alanine aminotransferase, T.bil: total bilirubin, CK: creatine kinase, CK-MB: creatine kinase- muscle/brain, CRP: C-reactive protein, IQR: inter-quartile range.

**Table 2:** Chief complaints of homeless patients.

Chief Complaints	*N*	%
Altered mental status	68	38%
Extremity pain	16	9%
General weakness	14	8%
Abdominal pain	12	7%
Multiple contusion	12	7%
Chest pain	9	5%
Fever, myalgia	7	4%
Dyspnea	7	4%
Dizziness	5	3%
Laceration	5	3%
Headache	4	2%
Back pain	4	2%
Facial pain	4	2%
GI bleeding	3	2%
Anxiety	3	2%
Eye problem	2	1%
Drug overdose	1	1%
Seizure	1	1%
Syncope	1	1%
Hallucination	1	1%
Total	179	

**Table 3:** Results of multivariate analysis for vitamin D deficiency in the homeless patients.

	Unadjusted Relative Risk *	95% Confidence interval	Adjusted Relative Risk *	95% Confidence Interval
Winter/spring	4.9	1.4–16.8	5.1	1.4–17.9
Hemoglobin	0.8	0.7–1.0	0.9	0.7–1.1
Albumin	0.6	0.2–1.2	1.0	0.3–2.7
CRP	1.1	1.0–1.2	1.1	0.9–1.2

* Calculated by log-binomial regression analysis.

## Supplementary Materials

The following are available online at https://www.mdpi.com/2072-6643/11/4/763/s1, Table S1: Characteristics of Homeless Whose laboratory tests were not done, Table S2: Characteristics of healthy control and homeless before propensity matching, Table S3: Characteristics of healthy same city (Seoul) control and homeless before propensity matching, Table S4: Characteristics of healthy national control and homeless after propensity matching, Table S5: Characteristics of healthy same city (Seoul) control and homeless after propensity matching, Figure S1: Emergency department treatment protocol for the homeless; EP: emergency physician.
