# Supplementary material for: High-Prevalence Vitamin D Deficiency among Korean Emergency Department Homeless, with a Comparison to a Healthy Korean Population
Source: Nutrients. 2019 Apr 1;11(4):763. doi: 10.3390/nu11040763 (PMC6521275; doi:10.3390/nu11040763)
Supplement: Supplementary file 1 [file nutrients-11-00763-s001.pdf]

Table S1. Characteristics of Homeless whose laboratory tests were not done.

Variables	*N* (%) or Mean ± SD
Total number	89
Age	51 ± 11
Male	82 (92%)
Alcohol-intoxicated state	64 (71.9%) *
Alcohol dependency	84 (94.4%)
Diabetes	13 (14.6%)
Hypertension	14 (15.7%)
Liver cirrhosis	8 (9.0%)
Malignancy	0 (0%)
Initial Presentation	
Musculoskeletal problem	72 (80.9%)
Acute pain	12 (13.5%)
Facial injury	2 (2.5%)
Gastrointestinal symptom	3 (3.4%)

*, P value less than 0.05 when comparing to the enrolled homeless; Student's T test or chi-square test were used.

Table S2. Characteristics of healthy control and homeless before propensity matching.

	Control *n* = 11067	Homeless *n* = 179	*p*-value
Male, *n* (%)	4707 (42.5%)	170 (95.0%)	<0.001
Age, mean ± SD	50 ± 17	52 ± 9	0.088
Hypertension, *n* (%)	2541 (23.0%)	21 (11.7%)	0.002
Diabetes, *n* (%)	918 (8.3%)	24 (13.4%)	0.020
Liver cirrhosis, *n* (%)	36 (0.3%)	16 (8.9%)	<0.001
WBC ($\times 10^3$/uL), mean ± SD	6.0 ± 1.7	8.0 ± 4.0	<0.001
Hemoglobin (g/dL), mean ± SD	14.0 ± 1.6	13.6 ± 2.1	0.001
BUN (mg/dL), mean ± SD	14 ± 4	14 ± 11	0.423
Creatinine (mg/dL), mean ± SD	0.8 ± 0.2	0.9 ± 0.7	0.018
AST (IU/L), mean ± SD	22 ± 14	86 ± 103	<0.001
ALT (IU/L), mean ± SD	21 ± 19	37 ± 37	<0.001

WBC: white blood cell, Hb: hemoglobin, BUN: blood urea nitrogen, AST: aspartate aminotransferase, ALT: alanine aminotransferase; Student's T test or chi-square test were used.

Table S3. Characteristics of healthy same city (Seoul) control and homeless before propensity matching.

	**Control** *n* **= 2304**	**Homeless** *n* **= 179**	*p***-value**
Male, *n* (%)	967 (42.3%)	170 (95.0%)	<0.001
Age, mean ± SD	48 ± 16	52 ± 9	<0.001
Hypertension, *n* (%)	460 (20.0%)	21 (11.7%)	0.008
Diabetes, *n* (%)	161 (7%)	24 (13.4%)	0.003
Liver cirrhosis, *n* (%)	4 (0.2%)	16 (8.9%)	<0.001 *
WBC (×10^3/uL), mean ± SD	5.9 ± 1.7	8.0 ± 4.0	<0.001
Hemoglobin (g/dL), mean ± SD	13.9 ± 1.6	13.6 ± 2.1	0.004
BUN (mg/dL), mean ± SD	14 ± 4	14 ± 11	0.141
Creatinine (mg/dL), mean ± SD	0.8 ± 0.2	0.9 ± 0.7	0.082
AST (IU/L), mean ± SD	22 ± 15	86 ± 103	<0.001
ALT (IU/L), mean ± SD	21 ± 22	37 ± 37	<0.001

WBC: white blood cell, Hb: hemoglobin, BUN: blood urea nitrogen, AST: aspartate aminotransferase, ALT: alanine aminotransferase; Student's T test or chi-square test were used; *, Fisher's exact test were used.

Table S4. Characteristics of healthy national control and homeless after propensity matching.

	**Control** *n* **= 358**	**Homeless** *n* **= 179**	*p***-value**
Male, *n* (%)	340 (95.0%)	170 (95.0%)	> 0.999
Age, mean ± SD	52 ± 9	52 ± 9	> 0.999
Hypertension, *n* (%)	82 (22.9%)	21 (11.7%)	0.003
Diabetes, *n* (%)	39 (10.9%)	24 (13.4%)	0.477
Liver cirrhosis, *n* (%)	5 (1.4%)	16 (8.9%)	<0.001
WBC (×10^3/uL), mean ± SD	6.4 ± 1.8	8.0 ± 4.0	<0.001
Hemoglobin (g/dL), mean ± SD	15.2 ± 1.3	13.6 ± 2.1	<0.001
BUN (mg/dL), mean ± SD	15 ± 4	14 ± 11	0.583
Creatinine (mg/dL), mean ± SD	1.0 ± 0.2	0.9 ± 0.7	0.048
AST (IU/L), mean ± SD	26 ± 14	86 ± 103	<0.001
ALT (IU/L), mean ± SD	27 ± 17	37 ± 37	<0.001

WBC: white blood cell, Hb: hemoglobin, BUN: blood urea nitrogen, AST: aspartate aminotransferase, ALT: alanine aminotransferase; Student's T test or chi-square test were used.

Table S5. Characteristics of healthy same city (Seoul) control and homeless after propensity matching.

	**Control** *n* **= 358**	**Homeless** *n* **= 179**	*p***-value**
Male, *n* (%)	340 (95.0%)	170 (95.0%)	> 0.999
Age, mean ± SD	52 ± 9	52 ± 9	0.900
Hypertension, *n* (%)	82 (22.9%)	21 (11.7%)	0.003
Diabetes, *n* (%)	33 (9.2 %)	24 (13.4%)	0.181
Liver cirrhosis, *n* (%)	1 (0.3%)	16 (8.9%)	<0.001
WBC (×10^3/uL), mean ± SD	6.2 ± 1.5	8.0 ± 4.0	<0.001
Hemoglobin (g/dL), mean ± SD	15.1 ± 1.2	13.6 ± 2.1	<0.001
BUN (mg/dL), mean ± SD	15 ± 4	14 ± 11	0.505
Creatinine (mg/dL), mean ± SD	1.0 ± 0.2	0.9 ± 0.7	0.027
AST (IU/L), mean ± SD	27 ± 26	86 ± 103	<0.001
ALT (IU/L), mean ± SD	28 ± 43	37 ± 37	0.022

WBC: white blood cell, Hb: hemoglobin, BUN: blood urea nitrogen, AST: aspartate aminotransferase, ALT: alanine aminotransferase; Student's T test or chi-square test were used; *, Fisher's exact test were used.

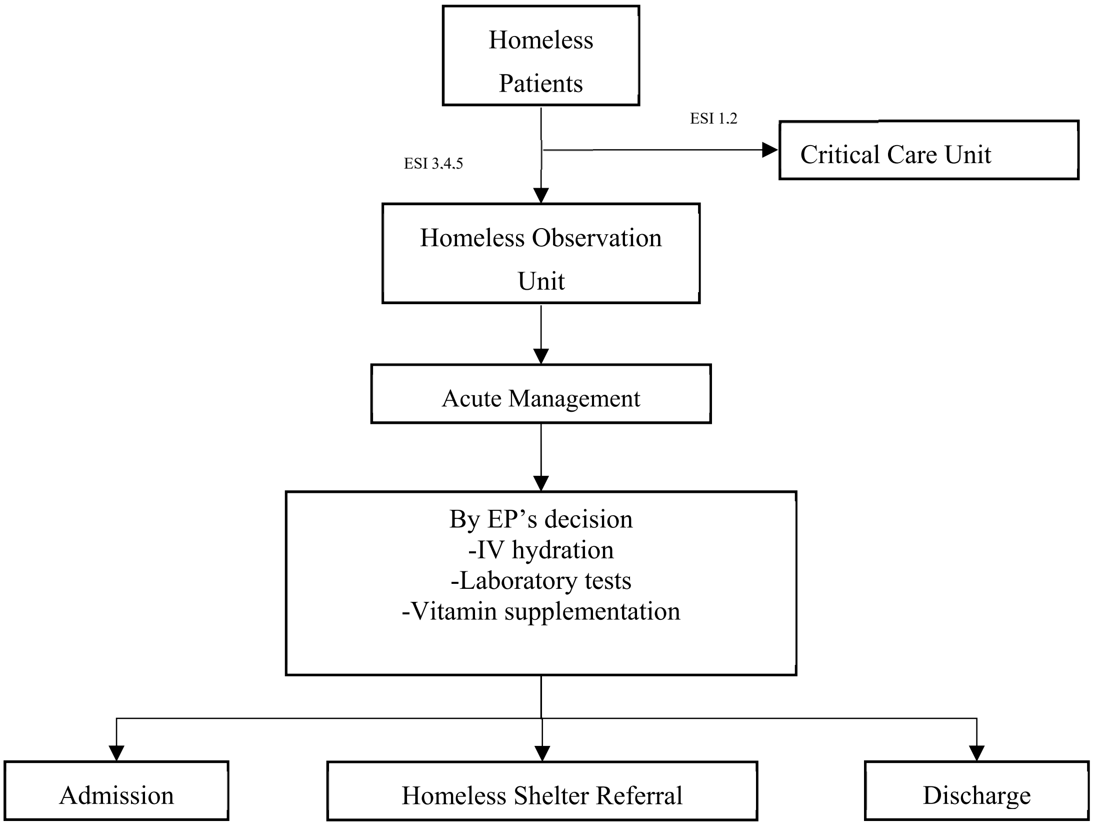

Figure S1. Emergency department treatment protocol for the homeless; EP: emergency physician.
